# A Unique Case of Heart Transplant and * Toxoplasma gondii* Infection in a Parasite- Seronegative Recipient: A Case Report

**DOI:** 10.2174/0118715303387775251028065817

**Published:** 2026-02-06

**Authors:** Lorenzo Giovannico, Thea Magrone, Anna Trentadue, Giuseppe Fischetti, Domenico Parigino, Ciro Isacco Gargiulo, Anna Maria Colacicco, Tomaso Bottio, Luigi Santacroce

**Affiliations:** 1 Cardiac Surgery Unit, Department of Precision and Regenerative Medicine and Ionian Area (DiMePRe-J), University of Bari Medical School, Piazza Giulio Cesare 11, Bari, Italy;; 2 Microbiology and Virology, Interdisciplinary Department of Medicine, School of Medicine, University of Bari, Bari 70124, Italy;; 3 Microbiology and Virology Unit, Policlinic University Hospital of Bari, Bari 70124, Italy

**Keywords:** Protozoan parasite, heart transplantation, toxoplasmosis, *Toxoplasma gondii*, acute renal failure, tracheotomy

## Abstract

**Introduction:**

The protozoan *Toxoplasma * (*T*.) *gondii* is responsible for toxoplasmosis, and this parasitosis represents a high risk in solid organ transplant procedures. In heart transplant patients, *T. gondii* infection is usually fatal since immunosuppressive drug therapy is administered to recipients.

**Case Presentation:**

A 62-year-old woman with severe biventricular dysfunction and seronegative for *T. gondii* underwent a cardiac transplantation from a donor seropositive for anti-*T. gondii* IgG antibody. The recipient exhibited post-transplant complications, including acute renal failure and difficulty in weaning from mechanical ventilation, ultimately requiring tracheotomy. The recipient underwent immunosuppressive pharmacological prophylaxis to prevent organ rejection. From a virological point of view, the recipient was monitored, and analyses performed on blood and serum revealed the presence of *T. gondii* DNA on blood. In addition, other viral and bacterial infections were observed. Afterward, molecular and anatomopathological investigations on cardiac biopsies were performed, and neither test revealed the presence of *T. gondii* DNA.

**Conclusion:**

On the one hand, prompt infection management and continuous monitoring are crucial to control viral and bacterial loads and, on the other hand, to optimise antimicrobial treatment, thus ensuring the gradual clinical stabilisation of the patient. Finally, it is important to highlight the need to review diagnostic screening protocols for organ donors to detect potential reactivation of microorganisms, viruses, and parasites that could be a fatal risk to recipients.

## 1 INTRODUCTION

*Toxoplasma* (*T*). *gondii* is a protozoan parasite responsible for toxoplasmosis, an infection that can affect individuals of any age or sex, transmitted through food contaminated with cysts or oocysts of the parasite [1]. Furthermore, it is important to note that transmission of *T. gondii* has been observed in patients undergoing solid-organ and haematopoietic stem cell transplantation [2, 3].

According to recent studies [4-6], the overall prevalence of donor-derived toxoplasmosis in heart transplant recipients remains low, although it is clinically significant due to its high morbidity and potential for fatality in seronegative recipients. Moreover, heart transplant recipients are highly at risk because *T. gondii* has a strong tropism to myocardial tissue. The reported clinical case highlights a rare but critical event, underlining the gaps in current screening and prophylaxis protocols. The clinical course of *T. gondii* infection in cardiac transplant recipients is generally favourable. However, in some cases, it can be fatal, particulary in immunosuppressed patients, as described by Wołyniec *et al.* [7] and Mastrobuoni *et al.* [8], who documented the sporadic detection of tissue cysts in myocardial biopsies. Finally, Hermanns *et al.* [9] reported a fatal case of cardiac transplantation from a *T. gondii* seropositive donor to a seronegative recipient, with the recipient dying three weeks post-transplant. Reactivation of latent toxoplasmosis in such patients may be triggered by immunosuppression or surgical stress, as noted by Akella *et al.* [3].

Although the parasite can localise in most tissues, serological analysis and Polymerase Chain Reaction (PCR) remain the cornerstones for accurate diagnosis [10]. This case report describes a rare case of detection of *T. gondii* DNA in the recipient's blood. The recipient was seronegative to anti-*T. gondii* antibodies (both IgM and IgG), whereas the donor was seropositive to anti-*T. gondii* IgG antibodies.

## 2 CASE PRESENTATION

We report the case of a 62-year-old woman who was followed up for severe aortic insufficiency (EROA: 0,30 cm^2^) with initial signs of left ventricular (LV) dilatation (LV diastolic diameter 58 mm) and underwent elective cardiac surgery.

After signing the informed consent, an aortic valve replacement was performed with a biological prosthesis number 23 through a ministernotomy at the fourth intercostal space, followed by the administration of blood cardioplegia directly into the coronary ostium. During weaning from extracorporeal circulation, hypokinesia of the right ventricle was observed on the transesophageal echocardiogram, with the ECG showing ST-segment elevation at aVF, D-II, and D-III. Extracorporeal membrane oxygenation (ECMO), using an arterial cannula in the ascending aorta, a vent in the cardiac apex, and a venous cannula in the left femoral vein, was placed. A coronary angiography was performed and showed a long spiral dissection from the proximal tract up to the posterior interventricular (IVP) branch. The spiral dissection extended to the distal section of the right marginal branch and the posterior descending artery. An intra-aortic balloon pump (IABP) was placed in the left femoral artery. Over the following days, the attempt to wean from mechanical assistance (ECMO and IABP 1:1) was ineffective, and treatment with continuous renal replacement therapy for oliguria was initiated.

The patient was transferred to the Department of Cardiac Surgery Ward of the Polyclinic Hospital of Bari to evaluate the potential inclusion in the emergency heart transplant list. Upon arrival, the patient was intubated in synchronized intermittent mandatory ventilation at 40%, responsive to simple orders, in V-A ECMO at 4.5 L/min and IABP 1:1, with dobutamine infusion 5 μm/kg/min and adrenaline 0.07 μm/kg/min. Chest radiography showed lung field congestion with a right pleural effusion. Transthoracic echocardiography revealed severe biventricular dysfunction (LVEF, 15%; TAPSE, 10 mm). Instrumental and laboratory tests and the patient's clinical conditions did not contraindicate admittance to the national emergency heart transplant list. The patient was transplanted (February 6^th^) seven days after the inclusion on the emergency heart transplant list. No noteworthy complications occurred. The ischemic time was 3 hours. The patient presented complications, including acute renal failure treated with haemodialysis and difficulty in weaning from mechanical ventilation, for which it was decided to wean *via* tracheotomy.

### 2.1 Clinical Microbiology Report at Admission and Therapeutic Procedures

The recipient's pre-transplant serological evaluation, carried out at the Microbiology and Virology Unit of the Policlinic Hospital of Bari, showed no antibodies against *T. gondii* (IgM and IgG) (Table **1**).

Post-transplant immunosuppressive therapy included mycophenolate mofetil and methylprednisolone. Tacrolimus was excluded due to acute renal dysfunction. Anti-thymocyte globulin was administered on postoperative days 1 and 4, respectively. In addition, prophylaxis included the administration of Valganciclovir plus Trimethoprim-Sulphamethoxazole (TMP-SMX), with a daily dose of 1 tablet containing TMP (80 mg) and SMX (400 mg) for the first 3 months following transplantation. However, this therapy was discontinued early due to acute cardiac damage, with plans to resume it during follow-up. 

On day 48 post-transplant, *T. gondii* DNA was detected in peripheral blood *via* PCR using the ELITeInGenius^®^ platform and ELITe MGB^®^ kit (ELITechGroup, a Bruker Company, Turin, Italy).This test amplifies a highly repeated genomic region (200-300 copies) specific to *T. gondii*.

**Fig. (1):**
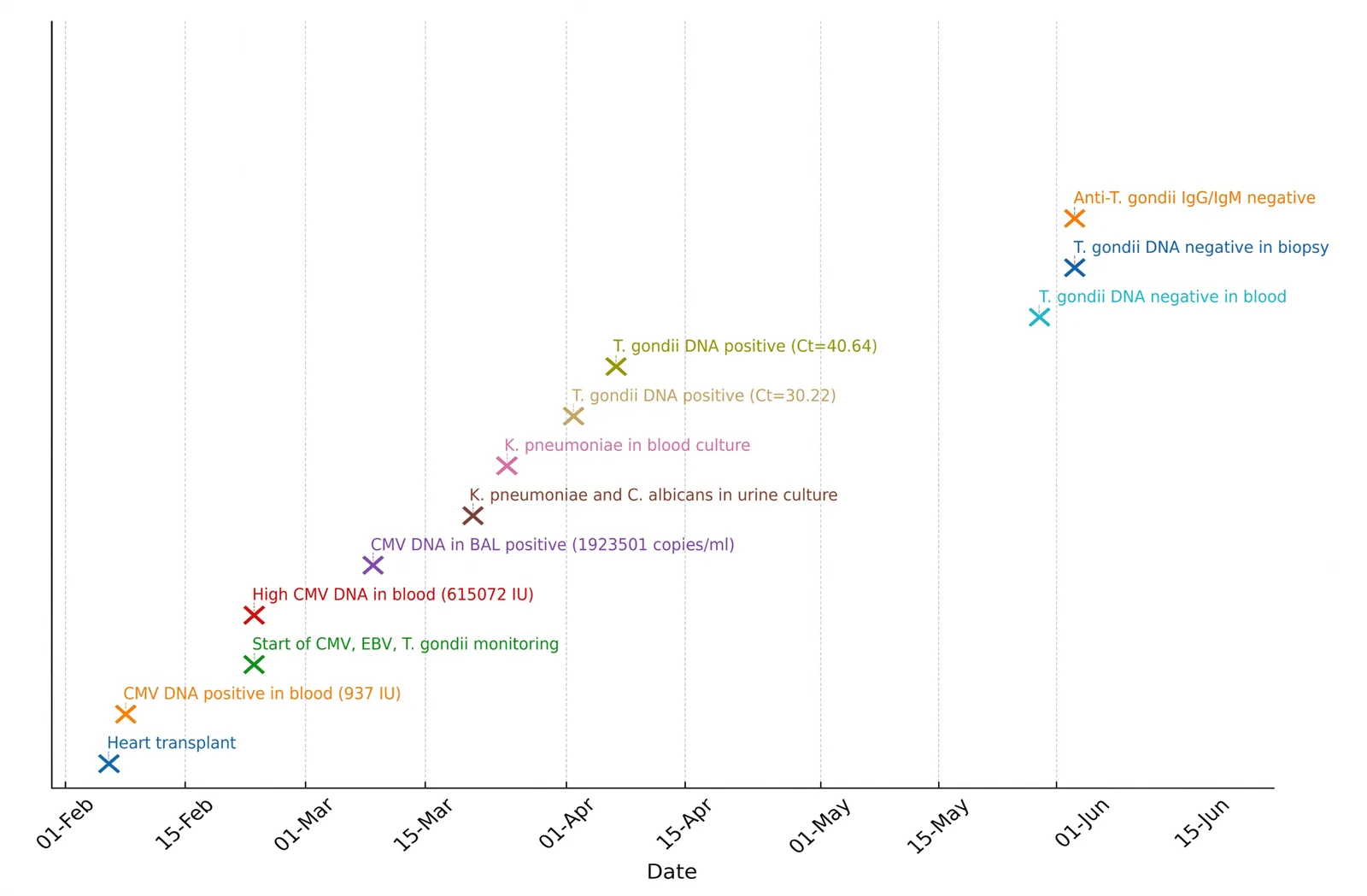
In this picture, the detection times for *T. gondii*, CMV, EBV, *Klebsiella (K.) pneumoniae* and *Candida (C.) albicans*, are illustrated.

The recipient's condition rapidly deteriorated, with headache, general malaise, fever, and respiratory distress. Moreover, 2 days after transplantation, Cytomegalovirus (CMV) DNA was detected in peripheral blood, with a viral load of 937 International Units (IU). CMV DNA was detected and quantified by means of PCR using CMV ELITE MGB**^^®^^** kit (ELITechGroup), according to the manufacturer's instructions.High CMV viral loads persisted until March 9^th^, when a markedly elevated level (1,923,501 genome copies/mL) was also detected in the bronchoalveolar lavage. By March 25^th^, CMV DNA levels had begun to decline, whereas *T. gondii* and Epstein-Barr virus (EBV) DNA remained undetectable. On April 2^nd^, *T. gondii* DNA appeared in peripheral blood with a cycle threshold (Ct) of 30.22, but by April 7^th^, the Ct had risen to 40.64, indicating decreased parasitaemia. Subsequent PCR tests for *T. gondii* DNA in peripheral blood, conducted on June 13^th^ and 17^th^, respectively, were negative. In addition, serological tests with chemiluminescence methods (CLIA) were conducted on June 17^th^ using the LIASON^®^ instrument (DiaSorin^®^ S.p.A., Saluggia, Italy) to assess the possible presence of antibodies directed against *T. gondii*. It is important to highlight the absence of seroconversion during follow-up, a phenomenon likely due to the severe immunosuppression*.* A post-transplant clinical timeline summarizes both microbiological and virological findings (Fig. ****1****).

In March, microbiological tests were performed on several samples, where *Klebsiella (K.) pneumoniae* and *Candida (C.) albicans* were observed in urine cultures, whilst *K. pneumoniae* was identified in blood cultures and central venous catheters, respectively. The isolated strain of *K. pneumoniae* was resistant to carbapenems (KPC), characterised by resistance to all antibiotics except ceftazidime associated with avibactam and aminoglycosides. On March 31^st^, the haematological picture showed anaemia, leukocytopenia, thrombo-cytopenia, and anisocytosis (Table **2**), likely due to systemic infection. Therefore, the recipient was transferred to the intensive care unit (ICU), where she underwent thoracotomy surgery and was discharged at the end of May due to overall clinical improvement.

 Clinical timeline of post-transplant *T. gondii* infection. 

On June 17^th^, after the ICU, dismissed recipient’s heart biopsy was performed, and the ventricular endomyocardial fragments showed a mild and focal interstitial lymphocytic infiltrate in the absence of myocyte damage. Finally, 1 month later, another heart biopsy was performed, and the result showed no changes related to acute cellular rejection, whereas diffuse hypertrophic-regressive changes in myocardiocytes were observed. In Figs. (**2A** and **B**), the myocardial sections are depicted.

In Fig. (**2A**), the myocardial biopsy performed on June 17^th^, stained with haematoxylin and eosin (H&E) at 10X magnification (original magnification: 400X), is illustrated. The ventricular endomyocardial fragments show a mild focal interstitial lymphocytic infiltrate in the absence of myocyte damage. The DNA PCR for the detection of *T. gondii* in myocardial tissue was negative. Heart muscle samples were fixed in 10% buffered formalin, embedded in paraffin, and stained with H&E.

In Fig. (**2B**), a histological section of an endomyocardial biopsy performed on July 17^th^, stained with immunohistochemistry for CD68, a macrophage/monocyte-specific marker, is shown at a magnification of 10X (original magnification: 400X). The biopsy revealed no evidence of acute cellular rejection; however, widespread hypertrophic-regressive changes of myocardiocytes were observed. The density of CD68-positive monocytes/macrophages is low, and the myocardial tissue appeared relatively preserved. The section was stained with H&E. CD68 monoclonal antibodies (Millipore^®^) were used at a dilution of 1:200 following a routine protocol (deparaffinisation, rehydration, antigen retrieval, inactivation of endogenous peroxidase, and blocking of non-specific reactions). The section was incubated with the primary antibodies for 12 hours at 4°C, followed by application of the labelled streptavidin-biotin-peroxidase complex (LSAB, Dako Corporation^®^) and visualised with diaminobenzidine (DAB kit; Dako Corporation^®^).

### 2.2 Donor

According to current organ donor screening guidelines, only IgG serology is required [11]. In this case, the donor tested was seropositive to anti-*T. gondii* IgG antibodies. The donor's medical history and condition showed a rather peculiar profile. The donor was a 76-year-old woman with a complex medical history, including arterial vasculopathy and prior surgical clipping of a cerebral aneurysm in 2016. She was a habitual smoker (approximately 10 cigarettes per day). Physical examination revealed cutaneous abnormalities, including keloids, petechiae, and ecchymoses. Cardiac assessment demonstrated mitral and tricuspid regurgitation. Laboratory investigations showed positivity for anti-hepatitis B virus (HBV) core IgG and anti-HBe IgG antibodies, whereas HBsAg was negative and HBV DNA was undetectable (<1 IU/mL). The donor was seropositive for CMV and *T. gondii* (Table **3**).

## 3 DISCUSSION

Toxoplasmosis is an infection that can lead to serious complications in immunocompromised individuals, including transplant recipients [12]. Transmission of *T. gondii* in solid organ transplant recipients is a rare but potentially life-threatening event, especially when a seronegative recipient receives an organ from a seropositive donor [2, 7, 9].

In this case, the donor is seropositive for anti-*T. gondii* IgG, whereas the recipient is seronegative for both IgG and IgM, an immune profile that significantly increased the risk of donor-derived toxoplasmosis [9]. As previously stated, since we do not have information on the presence of anti-*T. gondii* IgM donor antibodies, two hypotheses could be formulated. The first assumes that the donor is positive for both IgG and IgM antibodies directed against *T. gondii*. In this circumstance, an IgG avidity test could be essential to clarify whether the infection occurred before or after donation [13].

The second hypothesis is that the donor possessed only anti-*T. gondii* IgG antibodies, whereas IgM antibodies are negative. Current guidelines for donor screening do not recommend the determination of anti-IgM antibodies against *T. gondii* [11], and this is a limitation. On the other hand, high IgG avidity could suggest a past infection with a lower risk of transmission, whereas low IgG avidity could suggest a more recent infection with possible active transmission [13]. However, this test is not performed on the donor, and therefore, parasitic reactivation could not be ruled out entirely in this scenario. Furthermore, in both circumstances, transmission of tachyzoites *via* the transplanted organ could be considered plausible, especially in conditions of immunosuppression [14].

Therefore, this case highlights a clear need for careful consideration before discontinuing prophylactic regimens in high-risk recipients, along with the potential role of therapeutic administration alternatives when standard therapy is contraindicated. The diagnosis of toxoplasmosis in this patient is further complicated by the absence of a detectable serological response, probably attributable to severe immunosuppression due also to the post-transplant immunosuppressive therapeutic regimen.

As Nori and Ali stated in a previous study [15], this clinical picture exhibits unique features that indicate the immune system's inability, weakened by immunosuppression, to mount an adequate antibody response, even when the parasite is actively replicating and detectable by molecular methods such as PCR. Clinically, this case highlights the potential for serious consequences when the immune system is compromised and the crucial role of antibody production, in controlling and eliminating *T. gondii* infections. Regarding the prophylaxis initially administered, this is discontinued due to acute cardiac dysfunction. Given the timing of a PCR positive for *T. gondii*, discontinuation of TMP-SMX could have created a therapeutic window for the proliferation of the protozoan, particularly in an immunocompromised host. Literature supports that TMP-SMX is highly effective in preventing both reactivation of toxoplasmosis and primary infections, and its absence likely contributed to the outcome of this clinical case [16, 17]. This highlights the importance of balancing infection prevention with organ-specific complications and suggests that alternative prophylaxis or resumption could be implemented as soon as clinically feasible.

Moreover, the recipient experienced CMV reactivation and severe nosocomial infections caused by *K. pneumoniae* (carbapenems-resistant strain) and *C. albicans*. These complications are not unusual in immunocompromised patients and required aggressive, targeted therapy [18, 19]. However, these infections are also successfully resolved. Finally, another important feature of this case is the detection of *T. gondii* DNA by PCR in peripheral blood, but not in myocardial biopsies. Several mechanisms could explain these discrepancies: i) the uneven distribution of the parasite in the tissues, ii) the timing of sample collection, since PCR on blood is performed during the active phase of infection, whereas biopsies are performed later, during the recovery phase, and iii) the lower sensitivity of PCR in myocardial tissue compared to blood, possibly due to DNA degradation or sampling variability.

Based on the scientific literature and in view of the clinical case observed, we suggest that positive IgG detection in donors be carefully evaluated, indicating the need to promptly prepare and administer prophylactic therapy for recipients [20, 21]. In our opinion, despite the complexity of post-transplant outcomes, early molecular diagnosis and timely clinical intervention are fundamental strengths in achieving appropriate management to definitively stabilise the patient. Therefore, it is clear that accurate monitoring of reactivation of the infection in asymptomatic *T. gondii* recipients would be of great importance for the safety of organ donation. This clinical case highlights the importance of vigilant surveillance and flexible treatment strategies in high-risk transplant recipients.

## CONCLUSION

In conclusion, this case underscores the diagnostic and therapeutic challenges associated with donor-derived *T. gondii* infection in heart transplant recipients, particularly in the setting of a serological mismatch between a seropositive donor and a seronegative recipient. The detection of *T. gondii* DNA by PCR in peripheral blood, despite negative serology and biopsy findings, highlights the pivotal role of molecular diagnostics in identifying infections in immunocompromised patients. This case also highlights the importance of systematic donor and recipient screening, heightened clinical vigilance, and early molecular testing to ensure timely diagnosis and management of post-transplant toxoplasmosis.

## Figures and Tables

**Fig. (2):**
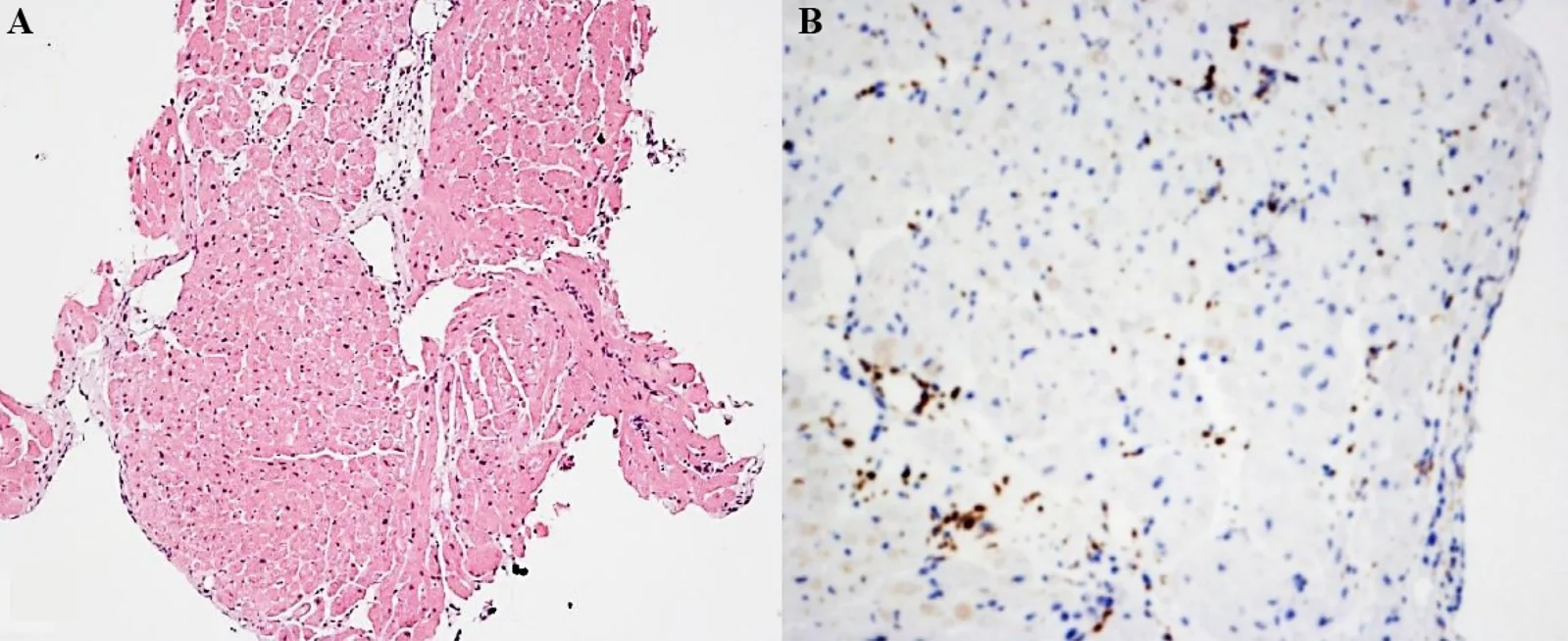
(**A, B**) Cardiac muscle tissue from an endomyocardial biopsies.

**Table 1:** Recipient's serological status before transplant.

-	Values	Reference Intervals		Results
*T. gondii* IgG Antibody	<3.00 IU/ml	<7.2 IU/ml	Negative	Negative
		≥7.2-8.8 IU/ml	Doubt	
		>8.8 IU/ml	Positive	
*T. gondii* IgM Antibody	<3.00 IU/ml	<6.0 IU/ml	Negative	Negative
		≥6.8.8 IU/ml	Doubt	
		>8.8 IU/ml	Positive	
CMV IgG Antibody	60.2 U/ml	<12 U/ml	Negative	Positive
		≥12-14 U/ml	Doubt	
		>14 U/ml	Positive	
CMV IgM Antibody	11.5 U/ml	<18 U/ml	Negative	Negative
		≥18-22 U/ml	Doubt	
		>22 U/ml	Negative	
EBV VCA IgG Antibody	483.0 U/ml	<20.0 U/ml	Negative	Positive
		≥20.0 U/ml	Positive	
EBNA IgG Antibody	83.4 U/ml	<5.0 U/ml	Negative	Positive
		≥5.0-20.0 U/ml	Doubt	
		>20.0 U/ml	Positive	
EBV VCA IgM Antibody	<10.0 U/ml	<20 U/ml	Negative	Negative
		20-40 U/ml	Doubt	
		>40 U/ml	Positive	
HSV1/2 IgG Antibody	>30.0 Index	<0.9 Index:	Negative	Positive
		≥0.9-1.1 Index	Doubt	
		>1.1 Index	Positive	
HSV1/2 IgM Antibody	<0.5 Index	<0.9 Index	Negative	Negative
		≥0.9-1.1 Index	Doubt	
		>1.1 Index	Positive	
VZV IgG Antibody	1935.0 mIU/ml	<135 mIU/ml	Negative	Positive
		≥135-165 mIU/ml	Doubt	
		>165 mIU/ml	Positive	
VZV IgM Antibody	7.1 Index	<9.0 Index	Negative	Negative
		≥0.9-1.1 Index	Doubt	
		>1.1 Index	Positive	

**Abbreviations:** CMV: Cytomegalovirus, EBNA: Epstein-Barr Nuclear Antigen 1, EBV: Epstein-Barr virus, HSV: Herpes Simplex virus, *T. gondii: Toxoplasma gondii*, VCA: Viral- Capsid Antigen, VZV: Varicella Zoster virus.

**Table 2:** Recipient's report before, during, and post-transplantation.

Serological Tests as of January 26^th^	
Vaccination coverage:	Rubella, Chickenpox, and Measles.
Contact immunization:	CMV, HSV1/2, EBV, VZV, Adenovirus, and Parvovirus B19.
Lack of antibody coverage:	*T. gondii.*
** Surveillance Results from February 8^th^**	
CMV DNA in blood samples:	From February 8th to March 23^rd^: Positive. From March 4^th^ to March 25^th^: Positive.
CMV DNA in BAL:	March 9^th^: Positive.
*T. gondii* DNA in blood samples:	April 2nd: Positive with Ct= 30.22. April 7^th^: Positive with Ct= 40.64. From June 13^th^ to June 17^th^: Negative.
*T. gondii* DNA in biopsy specimen:	June 17^th^: Negative. July 17^th^: Negative.
EBV DNA in blood sample:	Negative until May 7^th^.
IgG and IgM antibodies against *T. gondii*	June 17^th^: Negative.
** Microbiological Tests **	
Urine culture:	Positive for *K. pneumoniae* and *C. albicans*.
Blood culture:	March 25^th^ and March 26^th^: Positive for *K. pneumoniae*.
CVC:	March 27^th^: Positive for *K. pneumoniae*
** Haematological profile as of March 31^st^**	
Haemoglobin (Hb): 9.4 g/dl. Leukocytopenia: 2.840/µl. Thrombocytopenia: 166.000/µl. Anisocytosis: 17.2%.	

**Abbreviations:** **C. albicans:* *Candida albicans**, CMV: Cytomegalovirus, EBNA: Epstein-Barr Nuclear Antigen 1, EBV: Epstein-Barr virus, HSV: Herpes Simplex virus, *K. pneumoniae: Klebsiella pneumonia*, *T. gondii: Toxoplasma gondii*, VCA: Viral-Capsid Antigen, VZV: Varicella Zoster virus.

**Table 3:** Donor serological status.

-	Values	Results
*T. gondii* IgG Antibody	85.3 IU/ml	Positive
CMV IgG Antibody	72.6 IU/ml	Positive
EBV VCA IgG Antibody	483.0 U/ml	Positive
EBNA IgG Antibody	83.4 U/ml	Positive
HSV1/2 IgG Antibody	>30.0 Index	Positive
VZV IgG Antibody	1935.0 mIU/ml	Positive
Specific *Treponema pallidum* total antibodies	<0.10 AU/ml	Negative
*Strongyloides stercoralis* IgG	7.6 U	Negative

**Abbreviations:** EBNA: Epstein-Barr Nuclear Antigen 1, EBV: Epstein-Barr virus, VCA: Viral-Capsid Antigen, HSV: Herpes Simplex virus, *T. gondii: Toxoplasma gondii*, VZV: Varicella Zoster virus.

## Data Availability

The data supporting the findings of the study are available within the article.

## LIST OF ABBREVIATIONS

*C. albicans*: *Candida albicans*
CT: Cycle Threshold
CMV: Cytomegalovirus
EBNA: Epstein-Barr Nuclear Antigen 1
EBV: Epstein-Barr Virus
ECMO: Extracorporeal Membrane Oxygenation
HBV: Hepatitis B Virus
H&E: Haematoxylin and Eosin
HSV: Herpes Simplex Virus
IABP: Intra-Aortic Balloon Pump
ICU: Intensive Care Unit
IU: International Units
LV: Left Ventricular
*K. pneumoniae*: *Klebsiella pneumoniae*
PCR: Polymerase Chain Reaction
*T. gondii*: *Toxoplasma gondii*
TMP-SMX: Trimethoprim-Sulfamethoxazole
VCA: Viral-Capsid Antigen

## STANDARDS OF REPORTING

CARE guidelines were followed.
